# Association of microRNA-221/222 and -323-3p with rheumatoid arthritis via predictions using the human TNF transgenic mouse model

**DOI:** 10.1186/ar3660

**Published:** 2012-02-09

**Authors:** Ioannis Pandis, Caroline Ospelt, Niki Karagianni, Maria Denis, Martin Reczko, Carme Camps, Artemis Hatzigeorgiou, Jiannis Ragoussis, Steffen Gay, George Kollias

**Affiliations:** 1 Institute of Immunology, Biomedical Sciences Research Center "Alexander Fleming", Vari, Greece; 2 Center of Experimental Rheumatology, University Hospital Zurich and Zurich Center of Integrative Human Physiology, Zurich, Switzerland; 3 Biomedcode Hellas SA, Vari, Greece; 4 Institute of Molecular Oncology, Biomedical Sciences Research Center "Alexander Fleming", Vari, Greece; 5 The Wellcome Trust Centre for Human Genetics, University of Oxford, Oxford, UK; 6 Institute of Molecular Biology & Genetics, Biomedical Sciences Research Center "Alexander Fleming", Vari, Greece

## Background

MicroRNAs (miRs), a class of small non-coding RNA molecules, act as posttranscriptional regulators and are involved in a plethora of cellular functions. miRs have attracted a great deal of attention as potential therapeutic targets, as the sequence-specific mode in which they act, allows the simultaneous targeting of multiple target genes, often members of the same biological pathway(s) [1]. Previous studies have demonstrated that miRs are dysregulated and functionally involved in rheumatoid arthritis (RA) [2-9]. In this study we sought to identify novel miR associations in synovial fibroblasts (SFs), a key pathogenic cell type in RA [10,11], by performing miR expression profiling on cells isolated from the human TNF transgenic mouse model (TghuTNF, Tg197) [12] and patients biopsies.

## Materials and methods

miR expression in SFs from TghuTNF and WT control mice were determined by deep sequencing and the arthritic profile was established by pairwise comparisons. qRT-PCR analysis was utilised for profile validation, miR and gene quantitation in patient SFs. Dysregulated miR target genes and pathways were predicted via bioinformatic algorithms.

## Results

Deep sequencing demonstrated that TghuTNF-SFs exhibit a distinct pathogenic profile with 22 significantly upregulated and 30 significantly downregulated miRs (fold change>1.5, p-value<0.05). qRT-PCR validation assays confirmed the dysregulation of miR-223, miR-146a and miR-155 previously associated with human RA pathology, as well as that of miR-221/222 and miR-323-3p. Notably, the latter were also found significantly upregulated in patient RASFs, suggesting their association with human RA pathology. Bioinformatic analysis suggested Wnt/Cadherin signaling as the most significant pathway targets of miR-221/222 and miR-323-3p and CSNK1A1 and BTRC, the negative regulators of β-catenin, amongst predicted gene targets. qRT-PCR assays confirmed the downregulation of these genes in RASFs, validating our hypothesis that the newly identified miRs may function to modulate Wnt/Cadherin signaling.

## Conclusions

In this study, by performing comparative analyses between an established mouse model of arthritis and RA patient biopsies, we identified novel dysregulated miRs in RASFs potentially involved in pathways important for the pathogenic phenotype of these cells and highlighting the value of such cross-species comparative approaches [13].
